# Prediction of Esophageal Varices in Viral Hepatitis C Cirrhosis: Performance of Combined Ultrasonography and Clinical Predictors

**DOI:** 10.1155/2023/7938732

**Published:** 2023-09-15

**Authors:** Puwitch Charoenchue, Wittanee Na Chiangmai, Amonlaya Amantakul, Wasuwit Wanchaitanawong, Taned Chitapanarux, Suwalee Pojchamarnwiputh

**Affiliations:** ^1^Department of Radiology, Faculty of Medicine, Chiang Mai University, Chiang Mai 50200, Thailand; ^2^Department of Internal Medicine, Faculty of Medicine, Chiang Mai University, Chiang Mai 50200, Thailand

## Abstract

**Objectives:**

This study is aimed at evaluating the diagnostic performance of clinical predictors and the Doppler ultrasonography in predicting esophageal varices (EV) in patients with hepatitis C-related cirrhosis and exploring the practical predictors of EV.

**Methods:**

We conducted a prospective study from July 2020 to January 2021, enrolling 65 patients with mild hepatitis C-related cirrhosis. We obtained clinical data and performed grayscale and the Doppler ultrasound to explore the predictors of EV. Esophagogastroduodenoscopy (EGD) was performed as the reference test by the gastroenterologist within a week.

**Results:**

The prevalence of EV in the study was 41.5%. Multivariable regression analysis revealed that gender (female, OR = 4.04, *p* = 0.02), platelet count (<150000 per ml, OR = 3.13, *p* = 0.09), splenic length (>11 cm, OR = 3.64, *p* = 0.02), and absent right hepatic vein (RHV) triphasicity (OR = 3.15, *p* = 0.03) were significant predictors of EV. However, the diagnostic accuracy indices for isolated predictors were not good (AUROC = 0.63–0.66). A combination of these four predictors increases the diagnostic accuracy in predicting the presence of EV (AUROC = 0.80, 95% CI 0.69-0.91). Furthermore, the Doppler assessment of the right hepatic vein waveform showed good reproducibility (*κ* = 0.76).

**Conclusion:**

Combining clinical and Doppler ultrasound features can be used as a screening test for predicting the presence of EV in patients with hepatitis C-related cirrhosis. The practical predictors identified in this study could serve as an alternative to invasive EGD in EV diagnosis. Further studies are needed to explore the diagnostic accuracy of additional noninvasive predictors, such as elastography, to improve EV screening.

## 1. Introduction

Portal hypertension (PHT) is a common complication of cirrhosis. It leads to severe complications such as variceal bleeding and causes significant morbidity and mortality [1, 2]. Fifty percent of cirrhotic patients are at risk of developing esophageal varices (EV) [3–6], and a quarter of patients having EV intend to suffer from variceal bleeding over two years [4, 5]. About a twenty percent mortality rate is associated with each bleeding episode [5].

All patients with an initial diagnosis of cirrhosis are recommended to undergo screening esophagogastroduodenoscopy (EGD), the gold standard for the diagnosis of EV [3, 7–9]. However, EGD is an invasive procedure, associated with risk, and not tolerable in all patients [10]. Furthermore, it may not be available in a remote area without an endoscopist.

There are many indirect approaches to identifying EV. Hepatic venous pressure gradient (HVPG) is the gold standard hemodynamic measurement for assessing portal hypertension, predicting the risk of hepatic decompensation, and variceal treatment evaluation [11]. It can be used as an alternative tool for detecting EV [12]. Nonetheless, it is similarly invasive to EGD.

Noninvasive methods have gained attention as alternatives to invasive procedures like endoscopy. Few studies explore the clinical parameters, such as platelet count [13] and newer blood biomarkers, including serum laminin levels and serum hyaluronic acid [14]. However, the limitations of these tests are their accuracy in predicting EV and their availability. Clinical prediction rules such as FibroTest, APRI, and FIB-4, reflecting liver fibrosis, can help identify high-risk patients; however, they still do not directly predict EV.

Ultrasound (US) is one of the noninvasive modalities and has been developed and widely used for the follow-up of chronic liver diseases in identifying cirrhosis and hepatocellular carcinoma [15]. Combined clinical and ultrasound parameters such as platelet count and spleen diameter for predicting EV have good potential [16–22].

Transient elastography (TE, *FibroScan*®), measuring liver stiffness, has shown promise in predicting EV presence and severity. The Baveno VI criteria suggest the utilization of both TE with a liver stiffness measurement (LSM) value below 20 kPa and a platelet count exceeding 150,000 per milliliter (ml) to rule out high-risk varices [23]. Validation and further research are necessary to establish the accuracy and reliability of these noninvasive methods for EV screening in HCV cirrhosis patients. Liver and splenic stiffness measurements using TE or shear wave elastography (SWE) have been widely studied and may represent another potential predictor for EV [24–29]. However, the various machines' different values limit this method's global reproducibility.

The Doppler ultrasonography can be used for the hemodynamic evaluation of hepatic vessels like HVPG without invasiveness. Its measuring values are valid across the machines. The Doppler parameters showed good correlations with portal hypertension and liver stiffness measured by elastography and fibrosis staging obtained from a liver biopsy [30, 31].

Doppler ultrasonography can be a useful alternative for EV prediction because of its noninvasiveness, repeatability, and availability. We hypothesized that the hepatic vessels' Doppler sonography has high diagnostic accuracy in EV prediction in cirrhotic patients.

Previous studies on the Doppler ultrasound for predicting EV in cirrhotic patients were done, but their limited number of parameters and diagnostic performance for each parameter gave an inability to draw definitive conclusions [32–37]. The variability in results undermines the reliability of the Doppler ultrasound as a predictive tool for EV in cirrhotic patients. There was still no precision about choosing which branch of vessels to evaluate the hemodynamics. Moreover, no previous studies were done in all three vessels, including the portal vein, hepatic artery, and hepatic vein. Thus, these factors lead us to study more about the hemodynamic changes from Doppler ultrasound, a potential noninvasive method for predicting EV in a cirrhotic patient.

We believe that ultrasound could provide added diagnostic value in predicting EV. The objectives are (1) to evaluate the diagnostic performance of the Doppler ultrasonography and clinical predictors and (2) to explore the practical predictors predicting the presence of EV in viral hepatitis C cirrhotic patients.

## 2. Methods

Our prospective population-analog study with protocol identification number RAD-2563-07384 was approved by the Ethical Committee of Maharaj Nakhon Chiang Mai Hospital. Patients were recruited from the registered major project titled “Liver and Spleen Stiffness by 2-dimensional shear wave elastography to Predict Esophageal Varices in Patients with Hepatitis C virus-related cirrhosis” and coded as MED-2562-06671.

### 2.1. Study Population

**Figure 1:**
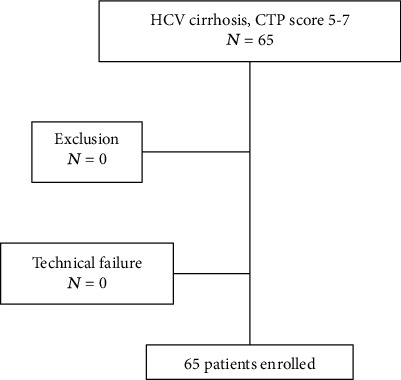
The study flow diagram shows no exclusion or technical failure.

Between July 2020 and January 2021, we consecutively enrolled 65 patients diagnosed with viral hepatitis C cirrhosis. All patients were 18 years or older and diagnosed based on a combination of physical, laboratory, and imaging examinations. Patients with a history of liver surgery such as transplantation, tumors, vascular interventions like transjugular intrahepatic portosystemic shunt (TIPS), congestive hepatopathy, or those who had undergone EV sclerotherapy were excluded to prevent the influence of confounding factors on the study results (Figure 1).

Patients who agreed to participate in our major research study were invited to join this study, and additional procedures involving the Doppler ultrasonography were explained, and written consent was obtained.

### 2.2. Clinical Data

Prior to conducting ultrasound and endoscopic examinations, demographic data such as age, gender, causes of cirrhosis, and laboratory and biochemical profiles were collected. The data collection process was conducted by a single research doctor who managed and recorded all information independently.

### 2.3. Ultrasonography

**Figure 2:**
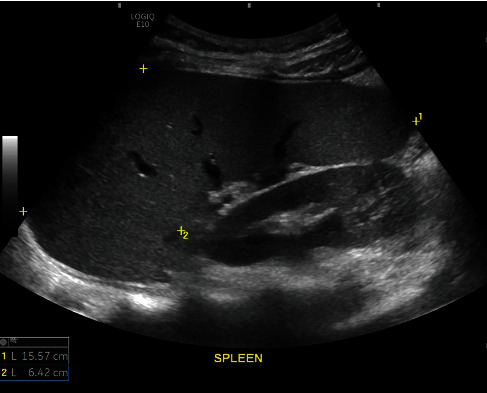
Grayscale US image shows the measurement of the splenic long and short axes.

Ultrasound examinations were performed on patients who had fasted for at least 6 hours. Two General Electric LOGIQ™ E10 machines equipped with curvilinear 1-6 MHz abdominal transducers were used. Patients were placed in the supine position, and the splenic long and short axes were measured using grayscale US (Figure 2). Subsequently, patients were positioned in the left lateral semirecumbent position with the right arm elevated above the head and instructed to hold their breath for several seconds. The ultrasound examination was performed using intercostal or subcostal approaches to achieve optimal imaging.

Parameters were measured using a maximal Doppler angle of 60 degrees, with time-averaged velocity calculated over two to three cardiac cycles. Three vessels were scanned, including the main portal vein (MPV), right hepatic vein (RHV), and hepatic artery (HA).

**Figure 3:**
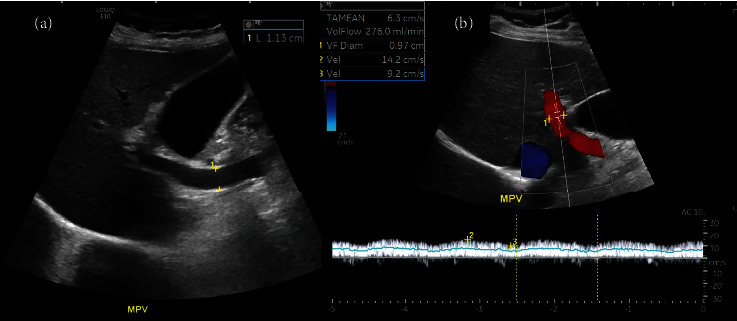
Assessment of the main portal vein: (a) grayscale US measuring a maximal diameter and (b) Doppler US measuring a maximal and minimal velocity and flow volume.

**Figure 4:**
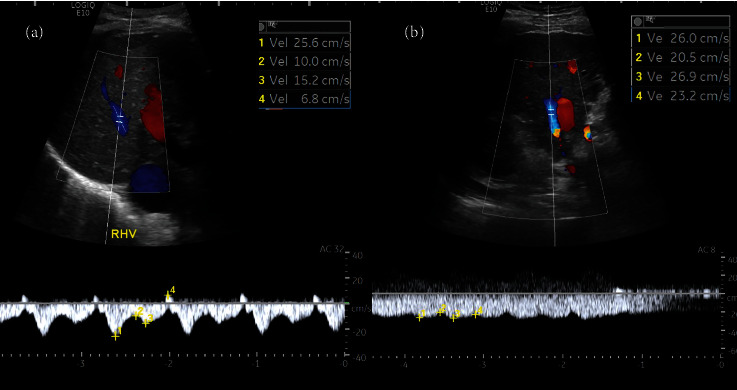
Assessment of the right hepatic vein for velocity and waveform type: (a) normal triphasic or (b) absence of triphasicity.

**Figure 5:**
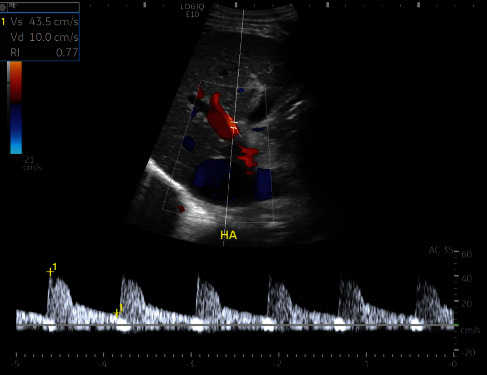
Hepatic artery assessment for PSV, EDV, and RI.

For the MPV, longitudinal scanning was performed approximately 2 cm below the bifurcation to obtain the maximal diameter (Figure 3(a)). The maximal and minimal portal vein velocities (cm/sec) and flow volume (ml/min) were automatically measured (Figure 3(b)). The RHV was measured approximately 4 cm from the insertion into the inferior vena cava to avoid the influence of cardiac reflux on waveform, maximal, and minimal velocities (cm/sec). The waveform was classified as normal triphasic or not (Figures 4(a) and 4(b)). The HA was scanned at the level of anterior crossing the MPV for peak systolic velocity (PSV) (cm/sec) and end-diastolic velocity (EDV) (cm/sec). The hepatic artery resistive index (HARI) was automatically calculated using the formula: HARI = PSV − EDV/PSV (Figure 5).

Patients who could not hold their breath or had no adequate acoustic window were excluded from the study. Two trained examiners, including one abdominal radiologist with five years of experience and one advanced body imaging fellow, performed the ultrasound scans separately. Each examiner completed the scan within 10 minutes and was blinded to the patients' clinical data throughout the study.

### 2.4. Endoscopic Evaluation

Within a month of the ultrasound examination, all patients underwent endoscopic testing performed by an experienced endoscopist with over ten years of experience. The endoscopist recorded the presence and severity of esophageal varices (EV), which were categorized as none (F0), small (F1), medium (F2), or large (F3) based on their appearance and size according to the Japan Research Society for Portal Hypertension [38]. The patients were then divided into two groups: those with no EV and those with any level of EV (F1-F3).

### 2.5. Data Analysis

We performed all statistical analyses using STATA® (version 16, StataCorp, Texas). We considered a *p* value of <0.05 to be significant. We expressed patients' characteristics as mean ± standard deviations, median ± interquartile range, or percentages as appropriate. To compare the groups with or without EV, we used *t*-test, Wilcoxon signed-rank test, or Fisher's exact test as appropriate with univariable analysis.

To assess the diagnostic accuracy of potential predictors for predicting the presence of EV, we calculated sensitivity, specificity, positive predictive value, negative predictive value, and the area under the receiver operating characteristic (ROC) curve using the results of endoscopy as the reference standard. We also used multivariable logistic regression to adjust the odd ratio of potential predictors, including sex, platelet count, splenic length, and absence of RHV triphasicity. We reported the prediction model's performance as the area under the ROC curve.

Furthermore, we evaluated the interobserver agreements of the Doppler US parameters using kappa statistics or intraclass correlation coefficient.

## 3. Results

Sixty-five individuals with cirrhosis due to viral hepatitis C were included in this study, with a mean age of 56.68 ± 8.4 years. The cohort comprised 28 women (43.08%) and 37 men (56.92%). All patients had mild cirrhosis, as evidenced by a median Child-Turcotte-Pugh (CTP) score of 5, which was similar across both groups. The cohort's average body mass index (BMI) was 25 ± 4.2 kg/m^2^.

### 3.1. Diagnostic Performances of the Doppler Ultrasonography and Clinical Predictors

The results of the study are presented in Tables 1–3.  Table 1 shows that 41.54% of patients had EV, with a significantly higher proportion of females than males (57.1% vs. 29.7%, *p* = 0.04). Patients with EV had lower platelet counts, with a median of 114,000 per milliliter.

Table 2 presents the univariable analysis of ultrasound indices, demonstrating that the splenic size and absence of normal right hepatic vein triphasic waveform differed significantly between EV and non-EV patients. The splenic length was significantly longer in patients with EV than those without (12.46 ± 2.44 vs. 10.54 ± 2.45, odds ratio 1.37, 95% CI 1.10-1.70, *p* = 0.003), as was the short axis (odds ratio 1.62, 95% CI 1.11-2.38, *p* = 0.008). Additionally, 22.22% of EV patients and 47.37% of non-EV patients had lost RHV triphasicity (*p* = 0.03).

Table 3 presents the diagnostic accuracy indices of four selected clinical and ultrasound parameters (gender, platelet count, splenic length, and absence of RHV triphasicity) for predicting EV, which demonstrated sensitivities ranging from 59.3% to 81.5% and specificities ranging from 57.4% to 68.4%. The areas under the ROC curves ranged from 0.63 to 0.66.

### 3.2. The Potential Predictors for Detecting the Presence of EV

In this study, we identified gender, platelet count less than 150,000 per ml, splenic length greater than 11 cm, and absence of right hepatic vein (RHV) triphasicity as potential predictors for the presence of esophageal varices (EV) due to their statistical and clinical significance.

We selected and limited the important variables, such as gender, platelet count, longest splenic length, and RHV triphasicity, using the backward elimination method. For instance, excluding a short splenic axis is employed to avoid having an excessive number of variables that could result in overfitting or impracticality in a clinical setting while ensuring the preservation of diagnostic capability. The measurement of the long splenic axis was chosen as it aligns with common practice in ultrasound assessment.

We performed multivariable logistic regression analysis to adjust for the coeffect of these predictors. We found that female gender (OR = 4.04, *p* = 0.02), platelet count less than 150,000 per ml (OR = 3.13, *p* = 0.09), splenic length greater than 11 cm (OR = 3.64, *p* = 0.02), and absence of RHV triphasicity (OR = 3.15, *p* = 0.03) were independently associated with the presence of EV (Table 4).

**Figure 6:**
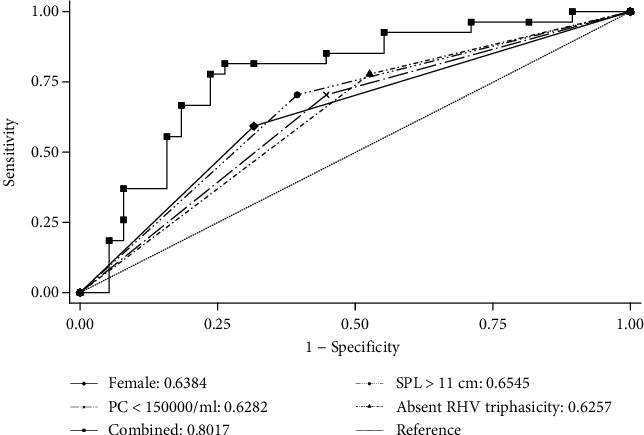
The receiver operating characteristic (ROC) curve shows the diagnostic accuracy of combined predictors for diagnosing esophageal varices (EV) compared to isolated parameters. The area under the curve (AUROC) for the combined predictor model was 0.80 (95% CI 0.69-0.91), indicating good diagnostic accuracy, while the AUROC for isolated parameters ranged from 0.62 to 0.65.

The predictive model for EV was calculated as *p*(EV) = −3.13 + 1.395057 (female) + 1.140788 (platelet < 150,000) + 1.007541 (spleen > 11 cm) + 1.436378 (absent RHV triphasicity). The area under the ROC curves in Figure 6 shows a significantly increased accuracy of the combined predictors with an area under the curve of 0.8017 (95% CI 0.69-0.91, SE 0.056), indicating good predictive ability.

### 3.3. Interobserver Agreement of the RHV Waveform Evaluation

The interobserver agreement was deemed good, as evidenced by a kappa index of 0.76.

## 4. Discussion

In our study, we conducted a prospective evaluation of clinical and ultrasound parameters to predict the presence of esophageal varices (EV) in patients with viral hepatitis C cirrhosis. Our multivariate logistic regression analysis identified female gender, platelet count, splenic length, and absence of the right hepatic vein (RHV) waveform as independent factors associated with EV. Our results showed that the prevalence of EV in HCV cirrhosis was 41.54%, which is consistent with previous studies [4, 5] indicating that approximately 50% of cirrhotic patients are at risk of EV development. However, we found that a higher proportion of females with mild cirrhosis had EV compared to males, which contrasts with previous research [39] showing no gender difference in EV prevalence. We suggest that further clinical or primary clinical science studies should investigate this gender effect.

In patients with cirrhotic liver disease, the platelet count is frequently observed to be reduced, a phenomenon attributed to two primary factors: the sequestration of platelets in the spleen and the diminished production of thrombopoietin. Moreover, the hepatitis virus has been shown to have a suppressive effect on bone marrow platelet production [40]. Notably, the platelet count represents a useful noninvasive predictor of esophageal varices (EV) in cirrhotic patients, with a correlation observed between platelet count and EV grading [41]. In particular, a platelet count below 150,000 per ml is associated with a high risk of EV [42]. This observation was confirmed in our study, wherein univariate and multivariate analyses both revealed a significant association between platelet count less than 150,000 per ml and EV.

The development of portal hypertension in hepatic cirrhosis is attributed to the constriction of the portal veins, leading to the accumulation of portal blood flow and increased resistance in the splenic venous outflow, consequently resulting in splenomegaly [35]. Our investigation focused on assessing the splenic size, and we found that a splenic length greater than 11 cm (sensitivity of 70.4%, specificity of 60.5%, and AUROC of 0.65) is indicative of the presence of EV in our study. These results are consistent with prior research, which has established that the extent of splenic enlargement is associated with the occurrence of EV [35, 37, 43–45].

Duplex Doppler ultrasound is widely recognized as the primary diagnostic modality for patients suspected of having portal hypertension [43]. Among the noninvasive techniques utilized in duplex Doppler ultrasound evaluation, the hepatic veins have emerged as an important focus of investigation. Typically, the waveform of the hepatic vein is classified into three patterns [46–49]. The first pattern is the triphasic pattern, which is typical of healthy individuals due to central venous pressure variations associated with the cardiac cycle [50, 51]. The second pattern is the biphasic pattern, which demonstrates no reverse flow and may exhibit decreased phasic oscillation, while the third pattern is the monophasic pattern, which displays a flat waveform. Previous studies have suggested classifying hepatic waveforms into six subtypes [52]. However, we found this classification system to be more complex and subject to greater inter- and intraobserver variability. Therefore, we utilized a simpler system that classified hepatic waveforms into three subtypes.

Previous studies have demonstrated a significant correlation between hepatic venous waveform alterations and hepatic dysfunction [47, 52–57]. These studies have shown that changes in hepatic venous waveform patterns can be indicative of underlying liver diseases, including cirrhosis. However, few studies have investigated the relationship between hepatic venous Doppler waveform patterns and esophageal varices (EV). Our study is aimed at addressing this gap and found that loss of the triphasic hepatic venous waveform is significantly associated with EV in cirrhotic patients with high accuracy. Consistent with previous studies, our findings suggest that the absence of the triphasic hepatic venous waveform may be a predictor of EV [58]. In addition, Baik et al. have reported that flattening of the hepatic venous waveform indicates a high likelihood of severe portal hypertension [32], which is consistent with our results. Our findings suggest that hepatic venous Doppler waveform analysis may be a useful noninvasive tool for identifying cirrhotic patients at risk of developing EV.

According to our result, the hepatic vein waveform classification was superior to the remaining Doppler parameters, which showed no significant correlation with the esophageal varices. These parameters included MPV diameter, maximal and minimal MPV velocity, MPV flow, maximal and minimal RHV velocity, HA PSV and EDV, and HARI. These parameters' measurements can be easily influenced by a slight error, such as the wrong location of blood flow detection, leading to inaccurate measurements, poor reproducibility, and limiting their clinical usefulness.

Regarding mean platelet volume (MPV) parameters, our study, consistent with previous research, found no significant association between EV and MPV [59–62]. Additionally, we observed increased hepatic artery resistance index (HARI) in cirrhotic patients but without significant correlation with EV, aligning with findings from previous studies [35, 37]. Our study suggests that HARI may not be a reliable predictor for EV in cirrhotic patients. As with other studies in the field, we found that the evaluation of hepatic vein waveform using a simple method allows for reproducibility and potential for widespread clinical use [52, 56, 58].

Our study has several limitations that should be taken into consideration. Firstly, our sample size was relatively small compared to some other studies. This led us to limit the parameters used in the prediction model. Thus, our results should be interpreted with caution and may require further validation in larger populations, including subgroup analyses for different etiologies and disease severities. Secondly, our patient population was limited to those with mild cirrhosis, as evidenced by the median CTP score of 5. Therefore, our findings may not be generalizable to patients with moderate or severe cirrhosis. Thirdly, our study only included patients with viral hepatitis C, which may limit the generalizability of our findings to other etiologies of cirrhosis that can also cause EV. Finally, although our method for evaluating hepatic vein waveform is relatively simple and reproducible, other noninvasive techniques or imaging modalities may be necessary for a comprehensive evaluation of portal hypertension in clinical practice.

In conclusion, our study found that a combination of clinical and Doppler ultrasound features can be used as an effective screening test for predicting the presence of esophageal varices (EV). Specifically, the combination of gender (female), platelet count (<150000 per ml), splenic length (>11 cm), and absent right hepatic vein (RHV) triphasicity, along with the Doppler assessment of the RHV waveform, showed higher diagnostic accuracy than isolated predictors alone. These findings suggest that the use of a combined screening approach could help clinicians identify patients at higher risk for EV, enabling earlier intervention and improved patient outcomes.

## Figures and Tables

**Table 1:** Patients' characteristics.

	EV *N* = 27 (41.54)	Non-EV *N* = 38 (58.46)	*p* value
Age	57.03 ± 7.06	56.42 ± 9.28	0.77
Gender			0.04
Female	16 (57.14)	12 (42.86)	
Male	11 (29.73)	26 (70.27)	
Weight (kg)	61.89 ± 13.84	65.13 ± 12.01	0.32
Height (cm)	156.79 ± 7.03	161.53 ± 10.17	0.05
BMI (kg/m^2^)	25.42 ± 5.59	24.93 ± 3.07	0.66
Hemoglobin (g/dL)	12.68 ± 1.60	13.30 ± 1.86	0.18
Platelet (median/ml)	114000	153000	0.009
Albumin (g/dL)	4.09 ± 0.59	4.24 ± 0.42	0.24
Globulin (g/dL)	4.13 ± 0.60	3.66 ± 0.58	0.003
ALP (U/L)	111.16 ± 39.89	87.89 ± 34.95	0.018
AST (U/L)	59.36 ± 41.32	57.86 ± 53.21	0.91
ALT (U/L)	47.84 ± 33.67	52.32 ± 38.46	0.64
TB (mg/dL)	0.99 ± 0.48	0.77 ± 0.51	0.10
DB (mg/dL)	0.46 ± 0.26	0.34 ± 0.23	0.06
PT (sec)	12.41 ± 1.27	11.43 ± 0.99	0.001
INR	1.13 ± 0.12	1.04 ± 0.09	0.001
PTT (sec)	34.37 ± 2.80	33.27 ± 2.95	0.15
Long axis spleen (cm)	12.46 ± 2.44	10.54 ± 2.45	0.003
Short axis spleen (cm)	5.71 ± 1.24	4.75 ± 1.50	0.008
MPV diameter (cm)	1.13 ± 0.15	1.06 ± 0.26	0.17
Max MPV velocity (cm/s)	18.52 ± 6.13	17.06 ± 5.25	0.31
Min MPV velocity (cm/s)	14.39 ± 4.14	13.33 ± 3.93	0.30
MPV flow (ml/min)	564.99 ± 310.34	533.53 ± 242.57	0.65
Max RHV velocity (cm/s)	36.61 ± 16.54	31.94 ± 10.68	0.17
Min RHV velocity, (cm/s)	14.81 ± 8.02	11.86 ± 6.24	0.09
RHV triphasicity, *n* (%)	6 (22.22)	18 (47.37)	0.03
HA PSV (cm/s)	54.85 ± 15.45	57.54 ± 18.72	0.55
HA EDV, cm/s	16.66 ± 5.49	19.16 ± 7.59	0.15
HA RI	0.69 ± 0.06	0.67 ± 0.08	0.12

Abbreviations: BMI: body mass index; ALP: alkaline phosphatase; AST: aspartate aminotransferase; ALT: alanine transaminase; TB: total bilirubin; DB: direct bilirubin; PT: prothrombin time; INR: international normalized ratio; PTT: partial thromboplastin time; MPV: main portal vein; RHV: right hepatic vein; HA: hepatic artery; PSV: peak systolic velocity; EDV: end-diastolic volume; RI: resistive index.

**Table 2:** Univariable analysis of ultrasound indices for the presence of esophageal varices.

Variables	EV	Non-EV	Odds ratio	*p* value
Long axis spleen (cm)	12.46 ± 2.44	10.54 ± 2.45	1.37 (1.10-1.70)	0.003
Short spleen (cm)	5.71 ± 1.24	4.75 ± 1.50	1.62 (1.11-2.38)	0.008
Loss RHV triphasicity	21 (77.78)	20 (52.63)	3.15 (1.04-9.54)	0.03
MPV diameter (cm)	1.13 ± 0.15	1.06 ± 0.26	5.00 (0.49-50.97)	0.17
Maximal MPV (cm/s)	18.52 ± 6.13	17.06 ± 5.25	1.05 (0.96-1.15)	0.31
Minimal MPV (cm/s)	14.39 ± 4.14	13.33 ± 3.93	1.10 (0.94-1.21)	0.30
MPV flow (ml/min)	564.99 ± 310.34	533.53 ± 242.57	1.00 (1.00-1.00)	0.65
Max RHV velocity (cm/s)	36.61 ± 16.54	31.94 ± 10.68	1.02 (0.99-1.07)	0.17
Min RHV velocity (cm/s)	14.81 ± 8.02	11.86 ± 6.24	1.06 (0.99-1.14)	0.09
HA PSV (cm/s)	54.85 ± 15.45	57.54 ± 18.72	0.99 (0.96-1.02)	0.55
HA EDV (cm/s)	16.66 ± 5.49	19.16 ± 7.59	0.94 (0.87-1.02)	0.15
HA RI	0.69 ± 0.06	0.67 ± 0.08	N/A	0.12

Abbreviations: MPV: main portal vein; RHV: right hepatic vein; HA: hepatic artery; PSV: peak systolic velocity; EDV: end-diastolic volume; RI: resistive index.

**Table 3:** Diagnostic accuracy indices of clinical and ultrasound parameters in predicting EV.

Predictors	Cut-off	Sensitivity	Specificity	PPV	NPV	AUROC
Sex	Female	59.3 (38.8-77.6)	68.4 (51.3-82.5)	57.1 (37.2-75.5)	70.3 (53.0-84.1)	0.64 (0.52-0.76)
Platelet count	150000/ml	70.4 (49.8-86.2)	55.3 (38.3-71.4)	52.8 (35.5-69.6)	72.4 (52.8-87.3)	0.63 (0.51-0.75)
Splenic length	10 cm	81.5 (61.9-93.7)	50.0 (33.4-66.6)	53.7 (37.4-69.3)	79.2 (57.8-92.9)	0.66 (0.55-0.77)
	11 cm	70.4 (49.8-86.2)	60.5 (43.4-76.0)	55.9 (37.9-72.8)	74.2 (55.4-88.1)	0.65 (0.54-0.77)
RHV triphasicity	Absent	77.8 (57.7-91.4)	47.4 (31.0-64.2)	51.2 (35.1-67.1)	75.0 (53.3-90.2)	0.63 (0.51-0.74)

**Table 4:** Multivariable logistic regression analysis adjusted by gender, platelet count, splenic length, and absence of RHV triphasicity.

Variables	Univariable		Multivariable	
	Odds ratio	*p* value	Adjusted odds ratio	*p* value
Female	3.15 (1.13-8.81)	0.03	4.04 (1.23-13.29)	0.02
Platelet < 150000/ml	2.93 (1.03-8.34)	0.04	3.13 (0.85-11.47)	0.09
Splenic length > 11 cm	3.64 (1.27-10.42)	0.02	2.74 (0.78-9.63)	0.12
Absent RHV triphasicity	3.15 (13.4-9.54)	0.04	4.20 (1.13-15.72)	0.03

## Data Availability

The unidentified clinical and ultrasound measurement data used to support the findings of this study were supplied by Puwitch Charoenchue under license and so cannot be made freely available. Requests for access to these data should be made to Puwitch C. (e-mail: Puwitch.c@cmu.ac.th).

## Abbreviations

EGD: Esophagogastroduodenoscopy
PHT: Portal hypertension
EV: Esophageal varices
HVPG: Hepatic venous pressure gradient
US: Ultrasound
TE: Transient elastography
SWE: Shear wave elastography
TIPS: Transjugular intrahepatic portosystemic shunt (TIPS)
MPV: Main portal vein
RHV: Right hepatic vein
HA: Hepatic artery
PSV: Peak systolic velocity
EDV: End diastolic velocity
HARI: Hepatic artery resistive index
ROC: Receiver operating characteristic
AUROC: Area under ROC curve.
